# Neighbourhood Environment and Stroke: A Follow-Up Study in Sweden

**DOI:** 10.1371/journal.pone.0056680

**Published:** 2013-02-15

**Authors:** Tsuyoshi Hamano, Naomi Kawakami, Xinjun Li, Kristina Sundquist

**Affiliations:** 1 Center for Community-based Health Research and Education, Organization for the Promotion of Project Research, Shimane University, Izumo, Japan; 2 Department of Environmental and Preventive Medicine, Shimane University School of Medicine, Izumo, Japan; 3 Waseda Institute of Sport Sciences, Waseda University, Tokorozawa, Japan; 4 Center for Primary Health Care Research, Lund University, Malmö, Sweden; 5 SPRC, Stanford School of Medicine, Stanford University, Stanford, California, United States of America; Morehouse School of Medicine, United States of America

## Abstract

**Background:**

In recent years, research on the association between physical environments and cardiovascular disease outcomes has gained momentum with growing attention being paid to Geographic Information Systems (GIS). This nationwide study is the first to examine the effect of neighbourhood physical environments on individual-level stroke, using GIS-based measures of neighbourhood availability of potentially health-damaging (fast food restaurants and pubs/bars) and health-promoting (physical activity and healthcare) resources.

**Methods:**

The study population comprised a nationwide sample of 2,115,974 men and 2,193,700 women aged 35–80 years who were followed between 1 December 2005 and 31 December 2007 in Sweden. Totally 42,270 first-ever strokes (both morbidity and mortality) were identified. Multilevel logistic regression models were used to estimate the association between neighbourhood availability of four different resources (fast food restaurants, pubs/bars, physical activity and healthcare) and individual-level stroke.

**Principal Findings:**

There were significant associations between neighbourhood availability of the four types of neighbourhood resources and individual-level stroke. The significant odds ratios varied between 1.06 and 1.12 for men and 1.07 and 1.24 for women. After adjustment for age, income, and neighbourhood-level deprivation, the increased odds remained statistically significant for neighbourhood availability of fast food restaurants in both men and women.

**Conclusions:**

Specific neighbourhood availability of resources were associated with individual-level stroke but most of these associations were explained by individual-level sociodemographic factors and neighbourhood-level deprivation.

## Introduction

During the last decade, there have been great efforts to identify the effect of neighbourhood environments on cardiovascular disease outcomes [1]. Most of previous studies have mainly focused on two broad types of neighbourhood environments: physical environments (e.g., air pollution, traffic noise) and social environments (e.g., socioeconomic deprivation, social cohesion) [2]–[6]. More recently, research on the association between physical environments and cardiovascular disease outcomes has gained momentum with growing attention being paid to Geographic Information Systems (GIS) [2], [7]. GIS allow us to combine a variety of data, and calculate availability of resources in order to characterize the physical environments in each neighbourhood [8].

Although previous research has analysed the possible associations between physical environments, assessed by GIS, and risk of coronary heart disease using multilevel analysis [7], very little is known about the association between physical environments and stroke. To date, there are limitations to existing work on GIS-based measures of physical environments and stroke. Specifically, previous research concluded that further work is needed to examine the independent effect of number of fast food restaurants on stroke after adjustment for individual-level risk factors in a multilevel fashion [9]. Only one study from the United States showed a significant association between the number of fast food restaurants and *neighbourhood* rates of stroke [9].Therefore, this nationwide study was designed to examine the effect of neighbourhood physical environments on *individual-level* stroke risk, using GIS-based measures of neighbourhood availability of potentially health-damaging (fast food restaurants and pubs/bars) and health-promoting (physical activity and healthcare) resources. The first aim of this nationwide multilevel study was to examine whether GIS-based measures of neighbourhood availability of health-damaging resources (fast-food restaurants, bars/pubs) or health-promoting resources (physical activity and healthcare facilities) was associated with individual-level stroke. The second aim was to test if these possible associations remained significant after adjustment for neighbourhood-level deprivation and individual-level sociodemographic characteristics.

## Methods

### Ethics statement

The study was approved by the Ethics Committee of Lund University. Data handling and analysis were performed by assigning serial numbers to data to preserve anonymity.

### Study population

The study population comprised a nationwide sample of 2,115,974 men and 2,193,700 women aged 35–80 years who were followed between 1 December 2005 and 31 December 2007 in Sweden. All individuals were included in a national Swedish research dataset, constructed at the Center for Primary Health Care Research at Lund University. This dataset contained nationwide individual-level medical diagnoses from the Swedish Hospital Discharge Register (obtained from the National Board of Health and Welfare) and the Cause of Death Register. These data were linked to the Swedish Population Register (census) data obtained from Statistics Sweden, the Swedish government-owned statistics bureau. The Population Register includes individual-level data on sociodemographic factors such as age and income.

### Stroke

The men and women were followed between 1 December 2005 (the start of follow-up) and 31 December 2007 for the outcome variable, i.e., first hospitalisation during the study period for stroke (both morbidity and mortality). The disease codes were based on the 10th version of the International Classification of Diseases (ICD-10) [10], and I60 to I69 were used to classify the outcome. In this study, men and women with pre-existing stroke were excluded. Totally 42,270 stroke cases (23,782 men and 18,488 women) occurred during the follow-up period.

### Health-damaging and health-promoting resources

Four categories of neighbourhood resources that could be regarded as potentially health-damaging or health-promoting were selected [7]. The four categories were fast food restaurants (e.g., pizzerias and hamburger joints), bars/pubs, physical activity facilities (e.g., swimming pools, gyms, ski facilities), and healthcare facilities (e.g., healthcare centres, public hospitals, dentists, pharmacies). Neighbourhood availability of the four categories was measured as counts per predefined administrative areas (Small Area Market Statistics, SAMS) by the use of GIS. We employed ArcGIS/ArcInfo 9.2 software from ESRI, which offers various ready-to-use spatial-analysis tools [11].

The ready to-use nationwide GIS dataset of business contacts (i.e., health-damaging and health-promoting resources) for November 2005 was provided to us by the Swedish company Teleadress [12]. Teleadress was created when the former government-owned company Telecom was divided into several subcompanies. It is a leading aggregator, processor and provider of Swedish contact information, delivering all available business telephone numbers, addresses and geographical coordinates in Sweden. The data included all business information in the Swedish Telephone Book (i.e., the Yellow Pages), in accordance with previous studies [13], [14].

SAMS units are predefined areas and were used as proxies for neighbourhoods, as has been done previously [5], [15]–[18]. Each SAMS unit has an average of about 1,000 residents. This study examined only those SAMS units that overlap with ‘localities’ or urban areas. In Sweden, ‘localities’ (which are defined by Statistics Sweden every fifth year) represent any village, town or city with a minimum of 200 residents and adjacent areas where the houses are no more than 200 m apart [19]. We chose to include only SAMS units overlapping with localities because more rural SAMS units have very few goods, services and resources. In 2005, 1940 Swedish localities were identified by Statistics Sweden. GIS were used to overlay the SAMS boundaries with the locality boundaries. Of a total of 9,617 SAMS units in Sweden, 7,945 overlapped with localities and were therefore selected. Together they accounted for 84% of the Swedish population. SAMS units with fewer than 50 people were excluded on the basis that they might yield unreliable statistical estimates in the calculation of the neighbourhood deprivation index. A final total of 7,033 SAMS units was included in the present study.

### Covariates

For gender, analyses for men and women were conducted separately. Age was categorised as 35–44, 45–54, 55–64, 65–74 and 75–80 years. Family income was categorised as empirical quartiles based on the distribution. The family income variable took the number of people in the family into account as well as the ages of the family members (children were given lower consumption weights than adults).

Neighbourhood-level deprivation was also included as a covariate because previous research has shown that neighbourhood deprivation is an important environmental disease determinant [15]. The neighbourhood deprivation index was constructed using 2005 census data provided by Statistics Sweden. A summary index was used to determine neighbourhood-level deprivation using the following four deprivation indicators for individuals aged 25–64 years (the working population): low educational status (<10 years of formal education); low income (income from all sources, including that from interest and dividends, <50% of the median individual income); unemployment (not employed, excluding full-time students, those completing compulsory military service and early retirees); and social welfare [18]. Each of the four variables loaded on the first principal component with similar loadings (+0.47 to +0.53) and explained 52% of the variation between these variables. A z score was calculated for each SAMS neighbourhood. The z scores, weighted by the coefficient for the eigenvectors, were then summed to create the index [20]. The index was categorized into three groups: below one standard deviation (SD) from the mean (low deprivation), above one SD from the mean (high deprivation), and within one SD of the mean (moderate deprivation). Higher scores reflect more deprived neighbourhoods.

### Statistical analysis

Age-standardised incidence proportions (proportions of subjects who became cases among those who entered the study time interval) were calculated separately for men and women by direct age standardisation using 10-year age groups, with the entire Swedish population of men or women aged 35–80 as the standard population.

Multilevel logistic regression models were created with incidence proportions as the outcome variables. These models are a good approximation of multilevel Cox proportional hazards models under conditions such as ours: a large sample size, low incidence rates, risk ratios of moderate size and relatively short follow-up [21]. The first model included neighbourhood availability of each of the four categories of neighbourhood resources in order to determine the crude odds ratio (OR) of stroke with 95% confidence interval (CI). The second model also included neighbourhood-level deprivation. The third model included neighbourhood availability for each resource, neighbourhood-level deprivation, and individual-level age and income. The analyses were performed using MLwiN [22]. A p-value of <0.05 was considered statistically significant.

## Results

Table 1 shows the number of subjects in the study population with availability/no availability of the four types of resources, number of stroke events and age-standardised incidence proportions (%) by neighbourhood deprivation and neighbourhood availability of the potentially health-damaging and health-promoting resources. Nearly half of the study population had at least one fast food restaurant in their neighbourhoods. Most people lived in neighbourhoods with no bars/pubs. Around 40% of subjects lived in neighbourhoods with availability to physical activity facilities or healthcare facilities. The age-standardised incidence of stroke increased with increasing neighbourhood deprivation. For the total study population, the incidence of stroke for men was 0.9% in low-deprivation neighbourhoods and 1.2% and 1.3%, respectively, in moderate- and high-deprivation neighbourhoods. The corresponding incidence proportions for women were 0.6%, 0.9% and 1.0%, respectively.

**Table 1:** Number of subjects with availability/no availability to resources, number of stroke events, and age-standardised incidence proportions (%) by level of neighbourhood deprivation and neighbourhood availability of potentially health-damaging and health-promoting resources.

	Men (n = 2,115,974)					Women (n = 2,193,700)				
	Number of subjects with availability/no availability to resources (%)	Number of stroke events	Neighbourhood deprivation			Number of subjects with availability/no availability to resources (%)	Number of stroke events	Neighbourhood deprivation		
			Low	Moderate	High			Low	Moderate	High
Neighbourhood resources		23,782	0.9	1.2	1.3		18,488	0.6	0.9	1.0
Fast food restaurants										
Availability	962,483 (45.5)	11,236	0.9	1.2	1.3	1,026,700 (46.8)	9,413	0.6	0.9	1.0
No availability	1,153,491 (54.5)	12,546	0.9	1.1	1.3	1,167,000 (53.2)	9,075	0.6	0.9	1.1
Bars/pubs										
Availability	268,552 (12.7)	3,202	0.9	1.2	1.3	296,407 (13.5)	2,845	0.6	0.9	1.0
No availability	1,847,422 (87.3)	20,580	0.9	1.2	1.3	1,897,293 (86.5)	15,643	0.6	0.9	1.1
Physical activity facilities										
Availability	867,451 (41.0)	10,055	0.9	1.2	1.3	904,607 (41.2)	7,950	0.6	0.9	1.1
No availability	1,248,523 (59.0)	13,727	0.9	1.2	1.3	1,289,093 (58.8)	10,538	0.6	0.9	1.0
Health care facilities										
Availability	816,483 (38.6)	9,780	0.9	1.2	1.3	881,646 (40.2)	8,364	0.6	0.9	1.0
No availability	1,299,131 (61.4)	14,002	0.9	1.2	1.3	1,312,054 (59.8)	10,124	0.6	0.9	1.1

Results of the associations between the four categories of neighbourhood availability and stroke are presented in Table 2. For men, there were statistically significant higher odds of stroke for those living in neighbourhoods with availability of fast food restaurants, bars/pubs, physical activity facilities and healthcare facilities in the unadjusted model (Model 1). The significant ORs varied between 1.06 and 1.12. After adjusting for neighbourhood-level deprivation (Model 2), neighbourhood availability of bars/pubs was no longer significantly associated with stroke. After further adjustment for individual-level age and income (Model 3), only neighbourhood availability of fast food restaurants continued to be significantly associated with higher odds of stroke.

**Table 2:** Associations between stroke and neighbourhood availability of potentially health-promoting and health-damaging resources.

	Men (n = 2,115,974)									Women (n = 2,193,700)								
	Model 1^a^			Model 2^b^			Model 3^c^			Model 1^a^			Model 2^b^			Model 3^c^		
	OR	95% CI		OR	95% CI		OR	95% CI		OR	95% CI		OR	95% CI		OR	95% CI	
Neighbourhood resources																		
Fast-food restaurants																		
Availability	1.07	1.04	−1.11	1.04	1.01	−1.07	1.02	1.00	−1.05	1.18	1.14	−1.22	1.13	1.09	−1.17	1.03	1.00	−1.06
No availability	1.00			1.00			1.00			1.00			1.00			1.00		
Bars/pubs																		
Availability	1.07	1.02	−1.11	1.03	0.99	−1.08	1.01	0.97	−1.05	1.16	1.10	−1.22	1.11	1.06	−1.17	1.00	0.96	−1.05
No availability	1.00			1.00			1.00			1.00			1.00			1.00		
Physical activity facilities																		
Availability	1.06	1.02	−1.09	1.04	1.01	−1.08	1.02	0.99	−1.05	1.07	1.03	−1.10	1.05	1.02	−1.09	1.02	0.99	−1.05
No availability	1.00			1.00			1.00			1.00			1.00			1.00		
Healthcare facilities																		
Availability	1.12	1.08	−1.15	1.07	1.04	−1.10	1.02	0.99	−1.05	1.24	1.19	−1.28	1.17	1.13	−1.22	1.03	1.00	−1.07
No availability	1.00			1.00			1.00			1.00			1.00			1.00		

OR odds ratio, CI confidence interval.

a Model 1 is unadjusted.

b Model 2 is adjusted for neighbourhood-level deprivation.

c Model 3 is adjusted for neighbourhood-level deprivation and individual-level age and income.

For women, a similar pattern was observed, with significantly higher ORs of stroke for those living in neighbourhoods with availability of fast food restaurants, bars/pubs, physical activity facilities and healthcare facilities (Model 1). The significant ORs varied between 1.07 and 1.24. These ORs remained significant after adjustment for neighbourhood deprivation (Model 2). After further adjustment for individual-level age and income (Model 3), only neighbourhood availability of fast food restaurants and health care facilities continued to be significantly associated with higher odds of stroke. For both men and women, the ORs that remained significant in the full model were only slightly increased and varied between 1.02 and 1.03.

There were no interactions between neighbourhood-level availability of the four resources and neighbourhood-level deprivation (data not shown).

## Discussion

To the best of our knowledge, no study has examined the effect of neighbourhood availability of potentially health-damaging and health-promoting resources on individual-level stroke risk in a multilevel fashion. The main finding of this study is that specific potentially health**-**damaging as well as health-promoting neighbourhood resources imply higher odds of stroke. Although the ORs of the associations are not large, our study included more than four million people aged between 35 and 80 years.

Our results are consistent with recent work from the United States, where an association between the number of fast food restaurants in the neighbourhood and stroke was found [9]. This association might be explained through high salt and caloric intake from fast food consumption leading to hypertension and obesity [9], [23], [24], which in turn may increase the risk of stroke. Our data did not, however, allow us to establish whether individuals with stroke consumed more fast food than those without stroke. This is a main limitation of the present study. Future work is needed to investigate dietary habits as potentially mediating pathways between availability of neighbourhood fast food restaurants and individual-level stroke.

The reason why high neighbourhood availability to healthcare facilities was associated with stroke is, however, uncertain. It might be explained by lack of timeliness [25]. This lack of timeliness can result in emotional distress, so that residents who live far from healthcare facilities may avoid seeking a medical treatment. As a result, the number of stroke patients in such neighbourhoods might be underestimated. To date, few studies have found barrier effects of spatial access to health care, with greater distance resulting in less health care utilization [26]. Although more research is needed to examine reasons for why such an association was shown in women, these results indicate that health policies might be focused on providing more equitable health care utilization.

These results extend previous findings on the association between GIS-based measures of physical environments and stroke. A study that examined the number of fast food outlets and stroke from the United States used census tracts as a proxy for neighbourhoods [9]. In the current study, however, SAMS units that has an average of about 1,000 residents was used to define a neighbourhood. SAMS units are much smaller than, for example, census tracts. This enabled us to assess availability in each individual's immediate neighbourhood, which increases the probability that the individuals were actually exposed to the potentially health-damaging and promoting resources in their daily lives. To date, there is little evidence on which neighbourhood definition is most appropriate in order to consider the effect of GIS-based measures of the physical environment on stroke. In addition, our study is the first of its kind, and our findings must be confirmed in other settings to provide more robust evidence in the formulation of efficient neighbourhood health policies.

The present study has several strengths. To our knowledge, this is the first large-scale study of an entire national population to examine the effect of potentially health-damaging and health-promoting neighbourhood resources on individual**-**level stroke in a multi-level fashion. The large-scale design allowed us to include 42,270 cases of stroke. Moreover, our study included adjustment for individual-level and neighbourhood-level covariates in the multilevel framework. This study also has certain limitations. First, our data did not allow for the assessment of other important risk factors for stroke, such as smoking, poor dietary habits, and physical inactivity. Second, we had no data on whether those individuals with stroke actually utilized the examined resources in their neighbourhoods. Third, the follow-up period was only two years. However, this means that the potential changes during the study period in neighbourhood availability of resources were most likely minor and that most people probably remained in their neighbourhoods.

## Conclusion

Our results suggest that specific neighbourhood-level resources are associated with individual-level stroke but that some associations seem to be explained by individual-level sociodemographic factors and neighbourhood-level deprivation. Caution is, however, warranted in the interpretation of these findings. Future studies should consider the actual use of neighbourhood resources in individuals with stroke and compare their use with healthy individuals.
